# Evaluating the Impact of a Dutch Sexual Health Intervention for Adolescents: Think-Aloud and Semistructured Interview Study

**DOI:** 10.2196/48453

**Published:** 2024-09-11

**Authors:** Gido Metz, Rosa R L C Thielmann, Hanneke Roosjen, Rik Crutzen

**Affiliations:** 1 Department of Health Promotion Care and Public Health Research Institute Maastricht University Maastricht Netherlands; 2 Soa Aids Nederland Amsterdam Netherlands

**Keywords:** web-based intervention, eHealth, engagement, potential impact, mixed methods, evaluation, acyclic behavior change diagram, web analytics, think-aloud method, web-based, user, chlamydia, behavior change

## Abstract

**Background:**

Engagement with and the potential impact of web-based interventions is often studied by tracking user behavior with web analytics. These metrics do provide insights into how users behave, but not why they behave as such.

**Objective:**

This paper demonstrates how a mixed methods approach consisting of (1) a theoretical analysis of intended use, (2) a subsequent analysis of actual use, and (3) an exploration of user perceptions can provide insights into engagement with and potential impact of web-based interventions. This paper focuses on the exploration of user perceptions, using the chlamydia page of the Dutch sexual health intervention, Sense.info, as a demonstration case. This prevention-focused platform serves as the main source of sexual and reproductive health information (and care if needed) for young people aged 12-25 years in the Netherlands.

**Methods:**

First, acyclic behavior change diagrams were used to theoretically analyze the intended use of the chlamydia page. Acyclic behavior change diagrams display how behavior change principles are applied in an intervention and which subbehaviors and target behaviors are (aimed to be) influenced. This analysis indicated that one of the main aims of the page is to motivate sexually transmitted infection (STI) testing. Second, the actual use of the chlamydia page was analyzed with the web analytics tool Matomo. Despite the page’s aim of promoting STI testing, a relatively small percentage (n=4948, 14%) of the 35,347 transfers from this page were to the STI testing page. Based on these two phases, preliminary assumptions about use and impact were formulated. Third, to further explore these assumptions, a study combining the think-aloud method and semistructured interviews was executed with 15 young individuals aged 16-25 (mean 20, SD 2.5) years. Template analysis was used to analyze interview transcripts.

**Results:**

Participants found the information on the Sense.info chlamydia page reliable and would visit it mostly for self-diagnosis purposes if they experienced potential STI symptoms. A perceived facilitator for STI testing was the possibility to learn about the symptoms and consequences of chlamydia through the page. Barriers included an easily overlooked link to the STI testing page and the use of language not meeting the needs of participants. Participants offered suggestions for lowering the threshold for STI testing.

**Conclusions:**

The mixed methods approach used provided detailed insights into the engagement with and potential impact of the Sense.info chlamydia page, as well as strategies to further engage end users and increase the potential impact of the page. We conclude that this approach, which triangulates findings from theoretical analysis with web analytics and a think-aloud study combined with semistructured interviews, may also have potential for the evaluation of web-based interventions in general.

## Introduction

### Background

The past few decades have witnessed a rise in the development and use of web-based interventions, and assessing engagement and the potential impact of such interventions typically necessitates using various methods [1]. For example, unobtrusive tracking of user behavior (eg, web analytics providing use data on page transfers or time on page) is extensively used as a method to measure engagement [2]. However, it has been argued that web analytics should not be used as the sole source of data in evaluating web-based interventions as they show how, but not why people behave as such [2-4]. This 1D measurement is particularly insufficient when potential impact is defined as the intervention users’ interaction with the relevant intervention content and application of it to their own specific context.

**Figure 1:**
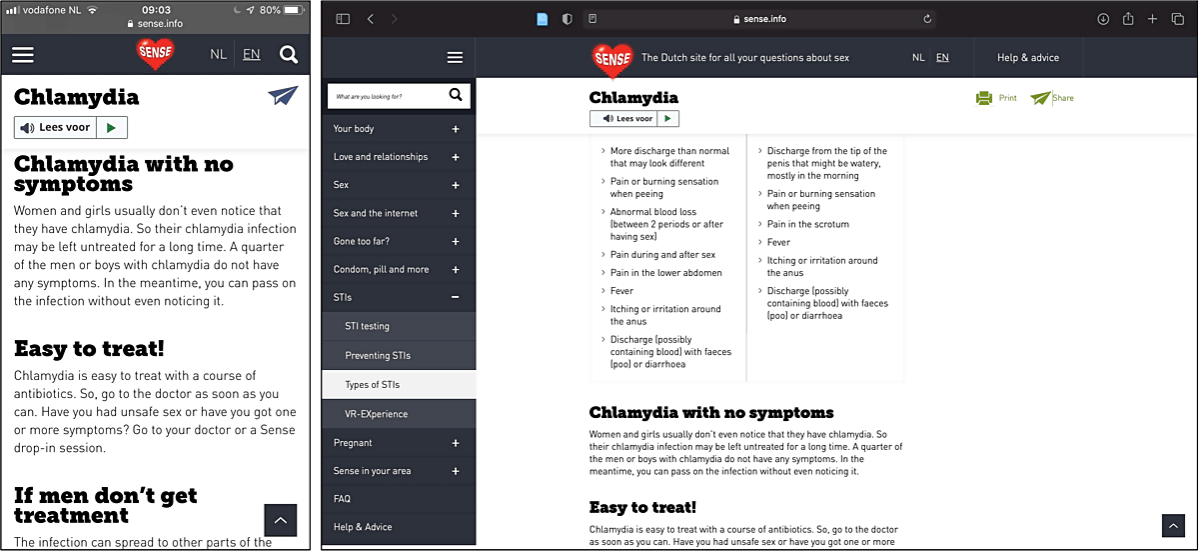
Screenshots of the chlamydia page of the Dutch sexual health intervention for adolescents (reproduced from Sense.info [5] with permission from Soa Aids Nederland) in 2020 (left: smartphone view, right: desktop view).

The overarching aim of this paper is to demonstrate how a mixed methods approach consisting of (1) a theoretical analysis of intended use, (2) a subsequent analysis of actual use, and (3) a further exploration of user perceptions, can be used to gain insight into engagement with and potential impact of web-based interventions. This demonstration takes place in the context of the chlamydia page of the Dutch sexual health intervention website Sense.info (Figure 1) [5]. This prevention-focused website serves as the entry point for young people aged 12 to 25 years in the Netherlands for information (and care if needed) regarding the whole spectrum of sexual and reproductive health. Sense.info has a wide reach: in 2021, the website received 4,358,543 visits from 3,917,923 unique visitors. Focusing on the Netherlands alone, Sense.info garnered 2,392,844 visits from 2,133,572 unique visitors (although it is possible that these visits were carried out by users outside this key audience, we mention here for reference that the Netherlands had 2,711,378 inhabitants between the ages of 12 and 25 years in 2021; [6]). In addition to the website, Sense.info actively uses TikTok [7] and Instagram [7] and hosts a podcast [8]. These channels serve both as independent means of engaging with their target audience and as a way of directing them to the website for more information.

In the following sections, we will outline the mixed methods approach and apply it to the Sense.info context. The first 2 phases of the mixed methods evaluation of Sense.info have already been carried out and reported elsewhere [9]. The results of these phases are outlined here because they form the basis of the third phase, which explores end user perceptions and is reported on in this paper.

### Analysis of Intended Use

The first phase involves a theoretical analysis of how the developers envisioned the use of the intervention and expected it to impact behavior change (ie, intended use). This analysis uses acyclic behavior change diagrams (ABCDs) to visualize the intervention’s active ingredients, underlying assumptions, and causal and structural relationships [10]. An ABCD consists of chains of seven links that illustrate (1) which behavior change principles have been applied—(2) taking into account the parameters for use—in (3) a specific application in an intervention, which (4) subdeterminants and (5) determinants have been addressed, and (6) which subbehaviors should be performed to achieve (7) the target behavior.

**Figure 2:**
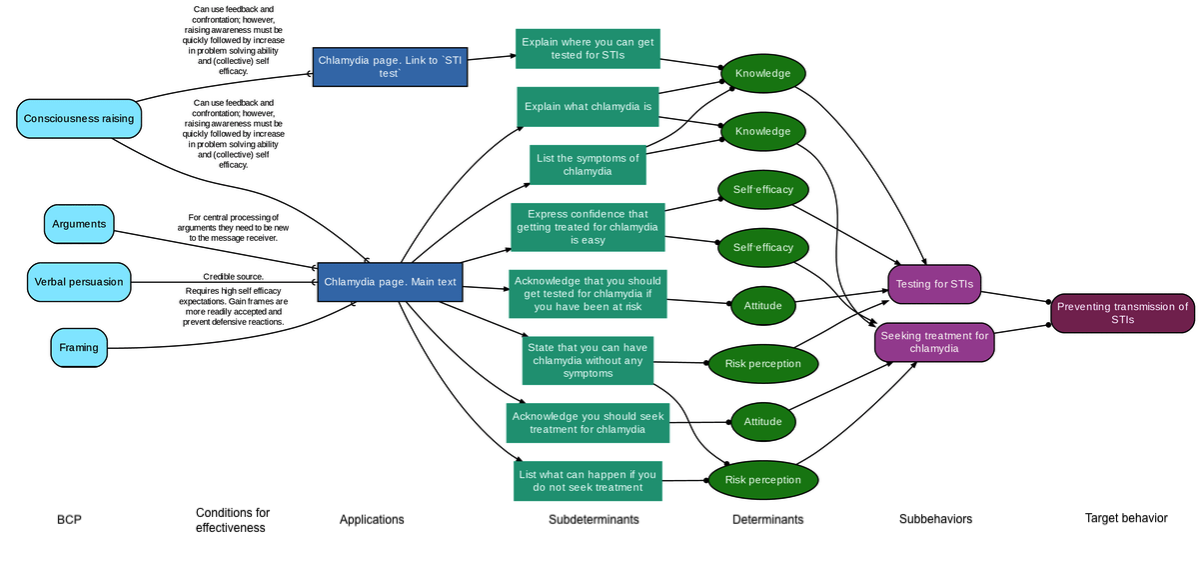
Extract of the ABCD for the Sense.info chlamydia page. The entire ABCD is available in the Open Science Framework Repository [13]. The ABCD visualizes which behavior change principles have been applied—taking into account the parameters for use—in a specific application in an intervention, which subdeterminants and determinants have been addressed, and which subbehaviors should be performed to achieve the target behavior. ABCD: acyclic behavior change diagram; BCP: behavior change principle; STI: sexually transmitted infection.

For the Sense.info chlamydia page, we labeled each element present on the page using the taxonomy of behavior change principles corresponding to the Intervention Mapping approach [11,12]. As shown in the ABCD excerpt for the chlamydia page in Figure 2, several behavior change principles, such as “consciousness raising,” were applied, targeting determinants such as risk perception and self-efficacy. This ultimately led to the subbehaviors of “getting tested” and “seeking treatment,” and to the target behavior “preventing transmission of sexually transmitted infections (STIs).” Based on our theoretical analysis, which highlighted the emphasis on testing on the chlamydia page, we assumed that the page would be most relevant to sexually experienced young people who may need STI testing.

### Analysis of Actual Use

Next, the actual use of the intervention is examined using web analytics. Specifically, we aim to identify use patterns that either match our expectations based on the analysis of intended use or reveal inconsistencies between intended and actual use. These findings will then lead to assumptions about the potential impact of the intervention, which will be further explored in the next phase.

Matomo, a web analytics tool, was used to study the use patterns of the Sense.info chlamydia page. In this paragraph, we outline the key data that formed the basis of our assumptions. Additional data can be found in a previously published paper [9]. Most visitors reached the page via a Google search (n=54,066, 54.4%), with queries particularly focusing on symptoms. Other visitors reached the page through Sense.info pages (n=37,287, 37.5%); mainly via a referral page with links to different STIs (<Types of STIs>; n=21,626, 58%), a page with a more general explanation of STIs (<What are STIs?>; n=3020, 8.1%), or via pages about specific STIs (<Gonorrhea>, n=1454, 3.9%; <Candida infection>, n=1342, 3.6%; and <Bacterial vaginosis>, n=1044, 2.8%). Although the chlamydia page is intended to motivate STI testing, a relatively small percentage (n=4948, 14%) of the 35,347 transfers from <Chlamydia> were to the STI testing page. The majority (n=14,493, 41%) of the transitions were to <Types of STIs>, followed by <Gonorrhea> (n=1979, 5.6%), <Genital warts> (n=1413, 4%), and <What are STIs?> (n=1413, 4%). Moreover, a high bounce rate (ie, the percentage of sessions in which visitors left Sense.info after viewing only the chlamydia page; 79%) and a relatively high exit rate (ie, the percentage of sessions that ended on that page; 69%) were reported.

These findings led to several preliminary assumptions about the use and potential impact of the page on STI testing behavior. Our first assumption was that visitors may have sought information about STIs out of curiosity, without an immediate need for STI testing. This would not necessarily be a negative outcome, as our goal was not to increase the transfer percentage to the STI testing page, but rather to study the potential impact of the page: did visitors in need of an STI test interact with the content relevant to them (eg, read information about symptoms, click on the link transferring them to the STI test page) and did they apply it (ie, take an STI test)? Visitors may have correctly concluded that an STI test was unnecessary or irrelevant to their situation.

The second assumption was that visitors had a need for an STI test after exposure to the intervention, but made an appointment with their general practitioner (GP) or at the Sense consultation hour (ie, consultation hours at municipal health centers specifically focused on the young individuals in the Sense.info target group) in a manner other than clicking on the link to the STI test page. In this case, the intervention, although undetected by web analytics, would have had an impact on the subbehavior of getting tested and ultimately on the target behavior of preventing STI transmission.

These first 2 assumptions did not necessarily imply that the low percentage of traffic to the STI test page was problematic. The third assumption, however, considered the possibility of a limited impact on the page by stating that visitors may have felt the need to test for STIs, but did not feel capable of getting tested and therefore did not click on the link to the STI test page. In such cases, additional efforts to increase self-efficacy might be needed.

### This Study: A Think-Aloud Study to Further Explore End Users’ Perceptions

The third and current phase of our approach is a think-aloud study combined with semistructured interviews. Our first aim is to shed more light on the assumptions about engagement and impact from the user’s perspective. The think-aloud method is useful for understanding cognitive processes and emotional reactions as participants navigate the intervention and view its content [1,14,15], and the semistructured interviews will allow participants to elaborate on the aspects mentioned during the execution of the think-aloud method. While the combination of a think-aloud method and semistructured interviews has been shown to be valuable [16,17], we believe that our think-aloud study will further benefit from the previous two analyses of intended and actual use, as these have provided us with assumptions about the potential impact of the intervention that can be further explored using the think-aloud method. The open nature of this method will allow assumptions to be confirmed or refuted, or new explanations to be added for the use of data. Our second aim is therefore to explore whether the triangulation of data from the analyses of intended use, actual use, and the exploration of end user perceptions is indeed valuable in assessing engagement and potential impact.

## Methods

### Ethical Considerations

This study was approved by the ethics review committee of the Faculty of Health, Medicine, and Life Sciences of Maastricht University (approval FHML-REC/2021/061). Participants signed informed consent and provided verbal consent reconfirmation before the start of the think-aloud procedure. Participants were reimbursed with a €25 (US $30 at a 1.20 conversion rate in June 2021) gift card after completion of the think-aloud and interview procedure. All transcripts have been deidentified. All materials used in this study—such as the participant information letter, informed consent form, and interview protocol—can be found in the Open Science Framework Repository [13]. The Consolidated Criteria for Reporting Qualitative Research (COREQ) checklist was used in the reporting of this study [18].

### Inclusion Criteria

Inclusion criteria were agreed upon with one of the developers of Sense.info, Soa Aids Nederland. Having had sexual interactions was a first criterion as it was expected that <Chlamydia> would be mostly visited by and relevant to sexually active people, and would also be most relevant to this group. Following reports on the age at which young people in the Netherlands start having sex [19], as a second criterion, we decided to include individuals aged 16-25 years. Third, in anticipation of the results of a scientific discussion in the STI prevention field about a possible narrowing of the target population for (asymptomatic) chlamydia testing and treatment due to recent findings about the relatively low severity of effects in most groups and antimicrobial resistance resulting from overtreatment with antibiotics [20,21], it was decided to include only individuals having heterosexual intercourse and cisgender individuals. As a final criterion, only people currently living in the Netherlands were included, as people living abroad cannot use all the services offered by Sense (eg, consultation hours). Visits to the website from abroad were also not included in our analysis of use data.

### Recruitment

Participants were recruited via a banner on Sense.info that appeared when website visitors spent more than 1 minute on the same page or when they transferred to another page on the website. The banner read: “Will you help us improve Sense.info? We are looking for young people to test this website. Reward: €25 gift card.” Clicking on the banner led to a page with general information about the study and a link to a short intake questionnaire (created in Formdesk) asking for sociodemographic information (age, being sexually active, education level, gender identity, sexual orientation, country of residence, country of birth, and country of parents’ birth). Only if the answers met the inclusion criteria, contact details (email address and phone number) were requested. Contact information was deleted upon completion of the study. If the inclusion criteria were not met, individuals were automatically redirected to a “thank you” page. Due to the sensitive nature of the inclusion criteria for gender identity and sexual orientation, individuals who did not meet these criteria were not automatically redirected to this page but received a personal email stating that they could not participate in this study but may be able to participate in a future study.

Study recruitment and execution began simultaneously. During the first weeks of recruitment, all eligible participants who had then signed up received an invitation email with a detailed information letter and a request to select a time slot in a “doodle,” a digital scheduling tool. A reminder was sent if participants did not respond within 5 working days. Based on the sociodemographic characteristics of the initial participants, purposive sampling based on age, education level, and gender identity was then used to reach as diverse a sample as possible and increase validity [22]. As a result, some individuals received both an invitation and a reminder, while others received only an invitation or no invitation at all. The latter was mostly the case for individuals who enrolled later in the process and had similar characteristics to those already recruited.

### Procedure

Data collection took place in May and June 2021 until data saturation was reached, defined as the point at which 3 consecutive interviews did not yield new relevant knowledge [23]. The procedure was carried out through videoconferencing using a professional Zoom (Zoom Video Communications) account. This approach made it easier to include people from all regions of the country. A technological advantage was that participants were able to share their screens, which allowed the researcher to unobtrusively observe their behavior. Appointments lasted approximately 1 hour. No one other than the participant and the researcher was present during the procedure.

GM (PhD candidate in Health Promotion, identifying as a man, educational background in social psychology and law [MSc, LLM], and trained and experienced in qualitative research methods) conducted the interviews. There was no relationship established with participants prior to study commencement, other than the email correspondence to explain the research project by means of an information letter and to set up a time and date for the think-aloud study. The participants knew that GM was a researcher at the Department of Health Promotion at Maastricht University and that the research was conducted independently from the partners at Soa Aids Nederland (developers of Sense.info), which was believed to make it easier for participants to talk freely about Sense.info. Participants were told beforehand that Maastricht University and Sense.info had the mutual goal of evaluating and optimizing Sense.info. No other characteristics about the interviewer were disclosed to the participants.

During the first minutes of the scheduled appointment, the researcher explained the study and the procedure following the information provided in the participant’s letter. Assuming that the use of screen-sharing options was more convenient on a desktop or laptop than on a smartphone or tablet, all participants were asked to use a desktop or a laptop. The page layout may vary slightly depending on the medium used, but the content remains the same (Figure 1 [5]). The participant then provided informed consent via a digital form (using Formdesk), after which the participant shared their screen and a practice think-aloud session began. The participant was asked to view the home page of the Trimbos Institute website [24] and perform a search task (find the Trimbos Institute’s phone number) while thinking aloud. This website dealt with health topics but was unrelated to the topics on Sense.info (no information about sexual health in general or chlamydia in particular).

After practicing and making sure the participant understood the procedure, the think-aloud procedure began. Screen and audio were recorded using QuickTime (Apple). Participants were asked to navigate the home page of Sense.info while thinking aloud. When they indicated that they had seen everything on the home page, the researcher asked them to search for the chlamydia page while thinking aloud and viewing the information as they would if they were alone. It was emphasized that they were free to read text, watch clips, and click on links to different pages (ie, there were no restrictions on what content they could choose or how long they could use it). If participants were silent for over 10 seconds, the researcher used the prompt “feel free to think aloud.”

When participants indicated that they had seen enough, the second part began: the in-depth interview using an interview protocol. The first questions were about the participants’ first impressions of the page. For example, they were asked what they thought of its appearance and comprehensibility and whether certain elements should be removed or added. Then, the researcher asked questions related to the determinants that were deemed relevant based on the analysis of intended use and remarkable use patterns found in the analysis of actual use. For example, the analysis of intended use showed that the behavior change principle “consciousness raising” was used to motivate treatment seeking for chlamydia by targeting the determinant of risk perception. This insight, combined with transfer rates to the STI test page, led to the question of whether the information was able to change participants’ beliefs about the effects of chlamydia.

### Analysis

Think-aloud sessions and semistructured interviews were transcribed verbatim and then sent to participants for confirmation of accuracy and approval for further use. Template analysis was used to analyze the transcripts because of its structured and stepwise approach to thematic analysis and its ability to incorporate both inductive and deductive themes [25]. The software used for the analysis was ATLAS.ti 9 (ATLAS.ti Scientific Software Development GmbH)*.* First, the coders (GM and RRLCT) read through and familiarized themselves with the data. They then conducted preliminary coding on a subset of the data, using both inductively and deductively derived themes, the latter based on the analyses of intended and actual use. For example, the ABCD created in the intended use analysis distinguished different subbehaviors (ie, getting tested, seeking treatment, using condoms, and notifying partners), for which we created corresponding codes. Based on the use patterns observed in the actual use analysis, we were interested in exploring why people would visit the page. Therefore, we created codes inductively, based on the reasons mentioned by participants (eg, when experiencing symptoms, when having had sex without a condom and suspecting that one’s partner might have chlamydia, when doing a school assignment on STIs, or when visiting out of interest). The same inductive approach was followed for the codes for barriers and facilitators related to the execution of the subbehaviors. The themes were then organized into clusters (eg, subbehaviors, barriers, facilitators, reasons for visiting the chlamydia page, usability, and reliability issues) and an initial coding template was defined. The initial template was applied to 10% of the data by the two coders independently, after which the coding was discussed and the template adjusted accordingly. This version of the template was then again independently applied to another 10% of the data set, based on which intercoder agreement was calculated using Krippendorff α [26,27] (α=.93). The template was applied to the entire data set by GM. Upon completion of this analysis, the results of this analysis were triangulated with the data from the intended and actual use analyses. The research team examined whether the results were consistent with the assumptions based on the intended and actual use analyses or whether the results suggested the possibility of a new explanation not covered by the assumptions.

## Results

### Participants

The digital intake questionnaire was accessible between April 30 and June 18, 2021, and was completed 238 times. A total of 85 submissions did not meet the inclusion criteria, resulting in a group of 153 eligible potential participants.

In total, 79 invitations were sent, of which 2 invitations were undeliverable due to an incorrect email address. A total of 24 appointments were made. Then, 33 reminders were sent, resulting in 2 additional appointments. Of the 26 appointments made, 4 participants canceled, 4 participants were not present at the appointed time and date, and 3 participants were found not to meet the age criteria at the beginning of the appointment.

The final sample consisted of 15 participants (mean age 20, SD 2.5 years), 10 of whom identified as female. All participants were still enrolled in education, except for 2 participants who, following the International Standard Classification of Education [28], completed lower secondary education and tertiary education (university of applied sciences). Two participants were enrolled in secondary education (1 lower secondary education and 1 upper secondary education), five participants in postsecondary nontertiary education, and six participants in tertiary education (3 university of applied sciences and 3 university). All but one of the participants were born in the Netherlands. Either one or both parents of 5 participants were born elsewhere, for example in Turkey, Morocco, or Surinam (these are also the most common migrant backgrounds in the Netherlands [29]).

### Perceptions About <Chlamydia>

#### Perceived Reliability

In total, 13 participants indicated that they found the information on <Chlamydia> reliable. Some reasons given were that Sense.info is used in education and the perception that it is government-funded. Three participants would have liked to see where the information came from to increase reliability. In addition, 5 participants explicitly stated that they found the information about chlamydia to be complete, but 4 participants mentioned that they would like to double-check information or seek additional information, mainly about the symptoms and consequences of chlamydia.

Maybe add the name of the person who created this, who contributed to the text or something. These are often people who have a medical degree ... Then it becomes more reliable. Or that’s how it looks then. But Sense does look like a good website. And I know that... at school they always say that it is a good source, that you can find very good information there.

P15

#### Reasons to Visit, Perceived Goals of the Page, and Expected Follow-Up Actions

All participants indicated that they would visit the page mainly if they had symptoms and wanted to investigate what STI they might have contracted (ie, as an information source for possible self-diagnosis). Related reasons were having had unsafe sex and having heard that a sex partner has chlamydia and therefore wanting to learn more about it and about the chance that they could have contracted it as well.

I think I would look it [<Chlamydia>] up when I would get symptoms after unprotected sex. And if I reached this [table of symptoms], I would have a look at the symptoms and if I were to recognize some of them, I would continue reading and then I would go see a doctor.

P12

When asked to elaborate on the goals they thought the page had, 8 participants mentioned providing information about chlamydia, and 6 participants thought the page aimed to refer people to an STI test. Perceived goals of the page and reasons to visit the page thus seemed to be consistent.

When asked about the specific actions they would take after viewing <Chlamydia>, 7 participants would do an STI test:

If I read this ... it says that not everyone shows symptoms, I would get tested then just to be sure you know, because maybe you don’t have any symptoms and then ... Yes, better safe than sorry.

P13

Six participants mentioned the possibility of consultation and an STI test at their GP and three participants talked about the option to do an STI test at the Sense consultation hours. Four participants mentioned partner notification as an action to be taken, and 3 participants mentioned buying and using condoms.

#### STI Testing: Barriers and Facilitators

Nine participants stated that learning about the symptoms and consequences of chlamydia would be a motivator to do an STI test, which seemed to be related to the perceived severity of the symptoms and consequences:

It’s a big step for you to get tested, but I think that if a woman finds out “Oh you can become infertile from this,” that they will think “Oh shit, let’s get tested very quickly.”

P07

This could be seen as an indication that providing information about the symptoms and consequences of chlamydia can influence knowledge, attitudes, risk perception, and intention to get tested. Three participants, however, overlooked the link to <STI test> during the think-aloud phase and stated that they did not see any information about STI testing on the page. Five participants thought it would be more convenient to place this link more prominently. When asked if they knew of other pathways on Sense.info to find information on STI testing, most responded in the affirmative.

This could also just be an information page. So only when I scroll down, I see: oh wait, you can do an STI test with them ... They do say ‘Sense consultation hours’ here, but if they made it a little bit clearer, for example by printing it in bold or whatever.

P03

There’s the search bar, an FAQ page on the left side, and a ‘Help & Advice’ page here, so there’s already three places where you can look it up in case you have a question, or you want an appointment or whatever.

P10

Participants also suggested options for interaction, such as a digital STI check, that would allow users to report their symptoms and then receive test recommendations and the option to schedule an STI test immediately. Two participants expected to find such a digital STI check by clicking on <STI test>, and they and four others recommended placing such a check on <Chlamydia>. Two participants stated that the concrete and personal advice from the STI check could help people decide what steps to take:

They could also make it interactive: “Do you have this symptom, this one, this one, yes,” then go to [sexual health center]. That might be a good addition for people so that they really know when you have to… so that you know right away which action is wise to take.

P09

In addition, another participant thought that such an STI check might lower the threshold for getting tested since people would not have to make a phone call:

Why is it only possible [to schedule an STI test] by phone? Because if they would... imagine they make some sort of little questionnaire, and based on that they will filter: “okay, these people are eligible to get tested.” Then they can also call those people or send a message saying “you have been approved, you can schedule a test” ... Because that would lower the threshold for scheduling a test.

P05

Some comments were made about the language used in relation to testing. One participant stated that the word “consultation hour” sounded “scary.” Another participant felt that the information did not encourage STI testing, but rather made it feel obligatory:

Are you worried, or have you had unprotected sex, don’t carry it around... Yeah... I might click on it, but I think this might be a bit scary. Because of the “don’t carry it around and get tested.” Might feel a bit, ehm, not threatening, but kind of like “you have to,” you know ... Yes, I think I would just have a sentence at the bottom or something like, “if you have any questions or if you are in doubt about anything, just get in touch with us,” something like that. Because I think you create a safer space then, like: “it’s okay, we’re here for you if you’re in doubt or if you think it’s scary.”

P07

The presence of a link to a personal story about chlamydia was evaluated positively by 7 participants, and such stories were seen as a good way to encourage STI testing by 2 participants. This may be through the mechanism of social identification. The young person in the story, who is similar to the reader, could become a role model by showing how they dealt with the same situation, which could motivate the reader to do the same.

Then it is a bit recognizable for yourself, that you realize: oh yes, it is indeed quite serious. Like you often do, when they put a quote somewhere. That way you can see it from the perspective of someone else who might have had the same, yes. So when you read that quote from somebody who’s has had the same thing, you think, maybe I should do it [testing] after all.

P06

Another participant also seemed to refer to the importance of social influence when they suggested providing insight into how many people get chlamydia.

Maybe they can do a subheading that says you don’t have to feel insecure or anything like that, or that it’s okay, maybe something like that. But I’m thinking about how that could be designed. Yes, here they describe the symptoms and everything, but ... Yeah, a subheading that it’s okay to get tested or that you’re not the only one or maybe add a graph about how many people get it [chlamydia]. So you don’t feel alone. I think the younger you are, the harder it is to take that step.

P14

## Discussion

### Principal Findings

The overall aim of this study was to demonstrate how a mixed methods approach using a think-aloud method combined with semistructured interviews and building on previously conducted analyses of intended and actual use, can be used to explain patterns in use data and to assess engagement with and potential impact of web-based interventions. This was studied in the context of the chlamydia page of the Dutch sexual health intervention Sense.info. Below, we first elaborate on the specific results concerning <Chlamydia> in relation to the 3 preliminary assumptions about the use, engagement, and potential impact of this page. After that, we share our methodological considerations about using think-aloud methods combined with other research methods to gain insight into web-based interventions in general.

Our first assumption was that visitors to <Chlamydia> were just looking for information about chlamydia without having the need for an STI test. However, participants mainly stated that they would visit the page with a self-diagnosis motive: investigating if they could have contracted an STI in response to showing symptoms or their partner having chlamydia. Although this finding might not immediately align with the relatively high bounce rates for the chlamydia page, other use data patterns do seem to show at least initial support for this self-diagnosis motive. Google search queries leading to <Chlamydia> mainly focused on symptoms, and parts of the visitors entering <Chlamydia> via other Sense.info pages had previously visited pages on other STIs. Most transitions from <Chlamydia> to other Sense pages were to <Types of STIs>, followed by pages about specific other STIs. These visitors may indeed have been trying to self-diagnose. Based on these findings, we might cautiously reject our first assumption and conclude that in general, visitors to <Chlamydia> have a need for an STI test.

In light of the above conclusion about the need for STI testing, it is relevant to note that participants generally found the information on <Chlamydia> to be reliable, but some indicated that they needed more information or wanted to verify information, for example, about symptoms or consequences of chlamydia. They stated that they would seek additional information elsewhere. Klawitter and Hargittai [30] found similar needs in their study about the perceived credibility of digital health information and termed it the consistency heuristic: users cross-reference various sources to determine whether the information is trustworthy. This finding may be one explanation for the relatively high bounce and exit rates of <Chlamydia>, although we recognize that several other explanations are possible. To help visitors find additional information, we recommend that the chlamydia page include links to reliable information elsewhere on the internet. In addition, the provision of information about (the credentials of the people) who compiled the page content may contribute to the perceived reliability.

Several participants explicitly mentioned the option of consultation and STI testing with their GP, in contrast to three participants who mentioned Sense consultation hours. This finding provides initial evidence to support the first part of our second assumption, that some visitors to <Chlamydia> feel the need for an STI test but make an appointment with their GP. This finding is consistent with data from the National Institute for Public Health and the Environment (RIVM) on STI testing in the Netherlands: two-thirds of all STI tests take place at the GP’s office; the rest at the sexual health centers, of which the Sense consultation hours are a part [31]. Regarding the second part of the assumption, that visitors make an appointment at the Sense consultation hour in a way other than by clicking on the link to <STI test>, we did indeed observe that participants overlooked the link but expressed that they knew of other ways on the website that lead to an appointment at the Sense consultation hour. Both the participants overlooking the link to the STI test page and participants getting an STI test at their GP might be explanations for the relatively high bounce rate found for the chlamydia page.

The third assumption was that visitors felt the need for an STI test but did not feel capable of getting tested and therefore did not click on the link to the page on STI testing. Nine participants stated that learning about the symptoms of chlamydia would encourage them to do an STI test. Following the extended parallel process model [32], we might conclude that the information on symptoms and consequences seems to be able to establish a threat high enough to lead to further cognitive processing. As depicted in the ABCD, this might impact risk perception of chlamydia and attitudes toward STI testing, which in turn could lead to STI testing. However, for a threat to lead to behavior change, there must be sufficient self- and response-efficacy-inducing information that leads individuals to adopt high perceptions of danger control (ie, high threat appraisal and high efficacy appraisal). Looking again at our ABCD for <Chlamydia>, we noticed that while the page provides messages that might influence self-efficacy regarding chlamydia treatment (“chlamydia is easy to treat with a course of antibiotics”), no such messages were present regarding STI testing. We did not find clear evidence of a lack of self-efficacy or response-efficacy among our participants regarding testing, although some participants mentioned certain barriers indicating that the information about testing did not always meet their needs (eg, no option for tailored advice, inappropriate location of the link to <STI test>, and not appealing use of language).

To help overcome the barriers mentioned above, it may be good to present STI testing as an easier and more natural step. Several options emerged from the interviews, for example, creating a digital STI check in which visitors are guided step-by-step in assessing whether they are at risk for an STI and can schedule a test immediately if needed. Moreover, personal stories seemed to induce motivation to get tested, potentially through the influence of social identification with the protagonist. Several theories indeed state that individuals are motivated to act in line with the behavior of influential others (social cognitive theory [33]) or with the norms of the group they identify with (social identity theory [34]). Based on these results, we suggest increasing the use of personal stories about chlamydia and STI testing, using the behavior change principle “modeling” [34]. This principle has been applied in both web-based and other interventions over the years and has shown success in initiating behavior change through heightened self-efficacy levels [35,36]. A suggestion given by a participant also seemed to touch upon social influence and modeling: they suggested providing information about the prevalence of chlamydia in young people to show visitors that they are not the only ones (potentially) having chlamydia. Similarly, the provision of numbers on how many visitors have done the digital STI test might motivate other visitors to do so as well.

### Practical and Scientific Implications

Specific insights into the engagement with and potential impact of the Sense.info chlamydia page were gained, as well as strategies to further engage end users and increase the potential impact of the page. For example, the inclusion of a digital STI check with tailored advice could be beneficial for those using the chlamydia page for self-diagnosis. In addition, the inclusion of role model stories may be a promising strategy to encourage STI testing among individuals who might be reluctant to test for STIs. These results are also deemed relevant for the optimization of other STI-related pages on Sense.info or other sexual health-related interventions.

Reflecting on the mixed methods evaluation process used, we conclude that it was valuable in assessing the potential impact and engagement of Sense.info and that it may also have value in evaluating other web-based interventions. The think-aloud method combined with semistructured interviews revealed usability issues, as well as psychological issues, for example, related to STI testing. The usefulness of the think-aloud method for usability issues was already known, as it is widely used in usability testing [37,38]. This is reflected in the methodology guidelines of Bonten et al [39], which mention the think-aloud method as a suitable method for the “design, development, and usability phase.” In addition to the importance of the think-aloud method in these early phases, the conclusion of this study is that this method also has value in what Bonten et al [39] describe as the “effectiveness or impact phase.” Since the guidelines also recognize mixed methods research for the effectiveness or impact phase, without specifying methods, we recommend the think-aloud method as a fitting method within such a design. This facilitates triangulation of data from several methods, which we see as essential. We [9], as well as others [1,2], argue that neither of the methods that we used should be used as the sole source of data to draw strong conclusions. For example, while a study of intended use sheds light on how theory should be or is applied in an intervention, it does not necessarily reflect how the intervention is used in daily practice [9]. Actual use data reveals how the intervention is used, but not why it is used that way [1,2]. A think-aloud method can provide several potential explanations for behavior, and we argue that it is likely most beneficial when building on results from studies examining intended and actual use. These prior findings can guide researchers in determining which aspects of the intervention require particular attention during the study.

### Limitations

A possible limitation of this study is that participants were asked not to use a smartphone but rather a computer (be it a laptop, desktop, or a PC with a webcam), as we expected that using screen-sharing options in Zoom would be most convenient on such devices. Although most young individuals between the ages of 12 and 25 years in the Netherlands have access to a mobile phone or smartphone (99.1%) [40], as well as to laptops or notebooks (96.1%) and PCs or desktops (63.4%), there may be a difference between access and preferred use. Most visitors to Sense.info use their smartphones. Therefore, future studies might consider allowing participants to participate using a device of their choice to create the most natural experience possible. Efforts to engage end users and consider their preferential use in the evaluation and subsequent optimization phase could lead to more equitable design [41,42].

A think-aloud study in which a participant is being observed will never be the exact same experience as an unobserved visit, and we need to take socially desired behavior into account. Furthermore, there are always multiple explanations possible for user behavior, and the explanations given by participants do not have to exclude other explanations. However, social desirability can never be ruled out in self-report research, and the think-aloud method is not unique in this respect. In the case of this study, we did not have the impression that participants behaved in a socially desirable way; they were open about several sexual themes including their own sexual health, and critically appraised the website.

Finally, there are two limitations regarding the recruitment of participants. First, potential bias as a result of recruitment cannot be ruled out. Participants self-registered for the study, which may have resulted in a pool of participants who were motivated and found it easy to talk about the issues at hand. This may have overlooked the insights of certain individuals who found it less easy to talk about these issues and therefore did not register for the study. A related issue is that there is no data on the characteristics of the real-life users of Sense.info. Whether a representative sample of Sense.info users was obtained, is therefore difficult to say. However, purposive sampling based on age, level of education, and gender was used to ensure that as diverse a group as possible was created from those who registered, and to reflect the diversity of the intended target audience of Sense.info. Second, only individuals having heterosexual intercourse and cisgender individuals were included in the study, anticipating the results of a scientific debate about narrowing down the target population for (asymptomatic) chlamydia testing and treatment to these groups. This would require a differentiation on the Sense.info website in the provision of chlamydia information based on sexual behavior, meaning that the current chlamydia page (with its emphasis on STI testing) would be maintained for cisgender people who have heterosexual intercourse, and a separate chlamydia page would be created for men who have sex with men and transgender people (with less emphasis on testing). During the design phase of the exploration of end user perceptions, the developers of Sense.info and the research team decided to focus on cisgender people who have heterosexual intercourse because, in the scenario described above, they would remain the group of interest for the current chlamydia page. After the completion of this study, it appeared that no changes in testing and treatment policy would be made in the near future, making a distinction unnecessary. In addition, the Sense.info use data did not allow analysis of use patterns based on sexual behavior, so it was not possible to verify whether use patterns could be explained by one group or the other. Including the entire Sense.info target group in the think-aloud study, regardless of sexual behavior, would therefore have been more consistent with the goal of exploring potential explanations for the use data. The views of individuals, such as men who have sex with men and transgender individuals, will therefore be included in future studies of the Sense.info chlamydia page.

### Conclusions

Using the Dutch sexual health intervention Sense.info, we demonstrated how a mixed methods approach consisting of a theoretical analysis of intended use, analysis of use data, and a think-aloud method combined with semistructured interviews can be used to gain insight into engagement with and potential impact of web-based interventions. This paper mainly discussed this approach in the context of Sense.info, but we believe it is valuable for evaluating web-based interventions in general.

## Abbreviations

ABCD: acyclic behavior change diagram
COREQ: Consolidated Criteria for Reporting Qualitative Research
GP: general practitioner
STI: sexually transmitted infection

## Data Availability

The data sets generated during or analyzed during this study (ie, interview transcripts) and all materials used (eg, participant letter and interview protocol) are available in the Open Science Framework Repository [13].
